# Prevalence and factors of sleep problems among Japanese children: a population-based study

**DOI:** 10.3389/fped.2024.1332723

**Published:** 2024-04-04

**Authors:** Asami Kuki, Ai Terui, Yui Sakamoto, Ayako Osato, Tamaki Mikami, Kazuhiko Nakamura, Manabu Saito

**Affiliations:** ^1^ School of Medicine, Hirosaki University, Hirosaki, Japan; ^2^ Department of Neuropsychiatry, Graduate School of Medicine, Hirosaki University, Hirosaki, Japan; ^3^ Department of Clinical Psychological Science, Graduate School of Health Sciences, Hirosaki University, Hirosaki, Japan

**Keywords:** sleep problems, 5-year-old children, population-based survey, neurodevelopmental disorders, JSQ-P, prevalence, socio-demographic, lifestyle

## Abstract

**Background:**

High prevalence of sleep problems in not only children with neurodevelopmental disorders (NDS) but also non NDS has been established. However, there are few studies that have looked into population-based and age-specific prevalence of sleep problems of children. Moreover, there are even fewer studies that have investigated the correlation of demographic and lifestyle-related factors affecting sleep problems in children. Considering these, the purpose of this study is to assess the correlation of the prevalence of sleep problems and selected socio-demographic and lifestyle-related factors in 5-year-old Japanese children in population-based study.

**Methods:**

Study children (SC) were recruited from two cohorts of the Hirosaki City 5-Year-Old Child Developmental Health Checkup Study. The first cohort consisted of 281 (162 males, 119 females) children recruited from 2014 to 2015, and the second cohort consisted of 2055 (1,068 males, 987 females) children from 2018 to 2019. In total there were 2,336 SC participants (1,230 males and 1,106 females). To determine the prevalence of sleep problems the Japanese Sleep Questionnaire for Preschoolers (JSQ-P) was utilized, and sleep problems are defined by a total score of ≥86. To determine socio-demographic and lifestyle-related factors affecting sleep, 10 factors (NDS diagnosis, birth month, childcare place, income, number of siblings, bedtime, waking time, sleeping hours, sleep onset delay, and screen time) were selected. Finally, to determine the correlation between prevalence of sleep problems and the selected demographic and lifestyle-related factors, data was analyzed using chi-square test.

**Results:**

The prevalence rate of sleep problems in 5-year-olds was 18% (369/2,055). Further, the prevalence of sleep problems was high in participants with ASD (50.4%), ADHD (39.8%), <2 million yen of income (30.5%), no siblings (24.2%), >22:00 of bedtime (30.7%), >7:30 of waking time (30.7%), <9 h of sleeping hours (25.3%), >30 min of sleep onset delay (35.3%), and ≥2 h of screen time (21.1%).

**Conclusion:**

The findings report 18% prevalence rate of sleep problems in 5-year-old children. Further, the findings establish a significant correlation of sleep problems and NDS, specific socio-demographic, and lifestyle-related factors. In considering the identified modifiable lifestyle-related factors contributing to sleep problems among the participants (i.e., bed/waking times and screen times), sleep programs to address these concerns are suggested.

## 1 Introduction

Sleep is a very important physiological function for children's health. Insomnia in early childhood and adolescence can negatively impact physical health, mental health, brain development, and externalizing behaviors. For example, insomnia has been reported to be associated with risk for weight gain (1), suicide (2), decreased hippocampal regional gray matter volume (3), or behavioral problems (4). Relations between sleep quality and obesity or school performance have been shown by several meta-analyses (5, 6). A recent meta-review showed that children's sleep has influences on their cognitive abilities, psychosocial health, adiposity, and risk-taking behaviors (7).

It has been reported that sleep problems in children appear early in their development. Approximately 20% of school-aged and adolescent children experience sleep difficulties (8, 9). Among preschoolers, problems with settling to sleep were present in 22% of 9-month-olds, 15%–20% of 1- to 2-year-olds, and 16% of 3-year-olds, and regular night waking were present in 42% of 9-month-olds, 20%–26% of 1- to 2-year-olds, and 14% of 3-year-olds (10).

Reports on the prevalence of sleep problems in children have varied by sleep measure and age. Takahashi et al. (11) reported that approximately 80% of Japanese 5-year-old children had sleep problems. Their study used the Children's Sleep Habits Questionnaire (CSHQ) as a sleep measure. Meta-analyses also showed that the prevalence of sleep problems in populations including preschoolers was as high as 54% (95% CI: 50%–57%) (12) and 38.9% (95% CI: 33%–45%) (13). The prevalence of sleep problems appears to vary due to differences in sleep measures and definitions of sleep problems.

Sleep problems are also associated with developmental characteristics. Previous reports showed that children with autism spectrum disorder (ASD) or attention deficit/hyperactivity disorder (ADHD) had a higher prevalence of sleep problems than children with no neurodevelopmental disorders (NDS) (14, 15). The prevalence of insomnia in children with ASD is 60%–86%, which is 2–3 times higher than that in children with non-NDS (14). In children with, 25%–50% experienced sleep disturbances (16).

Thus, it is known that many children, including those with non-NDS, as well as children with developmental characteristics, have sleep problems. However, previous studies had limitations such as the wide range of ages surveyed, different definitions and criteria for sleep problems depending on the sleep measure, and the difficulty in surveying children in the whole region. Therefore, the exact prevalence of sleep problems in 5-year-old preschoolers is unknown. In terms of research in Japan, there are very few population-based studies on children's sleep, and even fewer studies that focus on preschoolers.

In addition, the environment surrounding children's sleep can be influenced by lifestyle habits based on economic conditions, family composition, and culture. In fact, there are some reports that children's sleep conditions differ from country to country (17, 18). However, there are very few studies in Japan that have investigated various factors related to sleep other than diseases and disorders. Furthermore, as living environments have changed over the years, children's sleep has also changed over time (19), and parents and caregivers need new information about children's sleep.

The purpose of this study is to: First, assess the prevalence of sleep problems among 5-year-old Japanese SC participants. And second, determine the correlation between prevalence of sleep problems and selected socio-demographic and lifestyle-related factors affecting sleep in SC participants.

## 2 Methods

### 2.1 Study design

The research method was quantitative statistical analysis using epidemiological data such as sleep and lifestyle from the population-based Hirosaki Five-Year-Old Children Developmental Health Check-up Study (HFC study). First, to examine the prevalence of sleep problems, the data from 2,055 children over two years (2018 and 2019) in which all 5-year-olds were surveyed using the same sleep measure (Japanese Sleep Questionnaire for Preschoolers: JSQ-P) was obtained. Next, in order to investigate the correlation between developmental characteristics and sleep problems, as well as between sleep problems and selected socio-demographic and lifestyle-related factors, the data of 281 children from 2 years (2014 and 2015) of children at risk for NDS was added to increase the sample size.

### 2.2 Discussion on HFC study database

Hirosaki Five-Year-Old Children Developmental Health Check-up Study (HFC study) has been conducted since 2013 in Hirosaki and is a developmental survey targeting all 5-year-old children in Hirosaki City. The number of 5-year-old children surveyed each year is 1,200–1,300. The purpose of the research is the early detection and early support of NDS (20).

Hirosaki is a city in Aomori in Japan. It has approximately 164,000 people and 71,000 households (21). The population of under 15 years old is approximately 17,400 (22), the population of 5-year-old children in October 2023 is 1,135 (23).

The HFC study is conducted in two stages. The first stage is a primary survey of all 5-year-olds. A series of questionnaires were mailed by the City Health Center to parents or caregivers and kindergarten or nursery-school teachers, who completed the questionnaires and returned them to the health center. This questionnaire consists of several scales to measure characteristics of NDS and parenting stress, as well as a questionnaire on epidemiological information such as family structure and family income.

In the second stage, the detailed survey is conducted on children for whom developmental problems are suspected in the primary survey. The purpose of this stage is to perform multiple developmental tests and diagnose NDS. Detailed surveys and clinical diagnosis were conducted at the city health center. The detailed survey included examinations regarding cognitive and motor abilities, as well as structured interviews; Diagnostic Interview for Social and Communication Disorders (DISCO).

The clinical diagnosis was based on the results of detailed examinations and diagnosed ASD, ADHD, developmental coordination disorder (DCD), and intellectual disability (ID) according to Diagnostic and Statistical Manual of Mental Disorders—5th edition (DSM-5) criteria. Autism Diagnostic Observation Schedule 2nd edition (ADOS-2) was added for the diagnosis of ASD, and the guidelines of the European Academy of Pediatric Disorders were added for the diagnosis of DCD. ID was defined as intelligence quotient (IQ) was below 70. The diagnosis was determined through consensus at a multi-disciplinary conference.

(Refer Supplementary Table A).

### 2.3 Sampling

**Figure 1:**
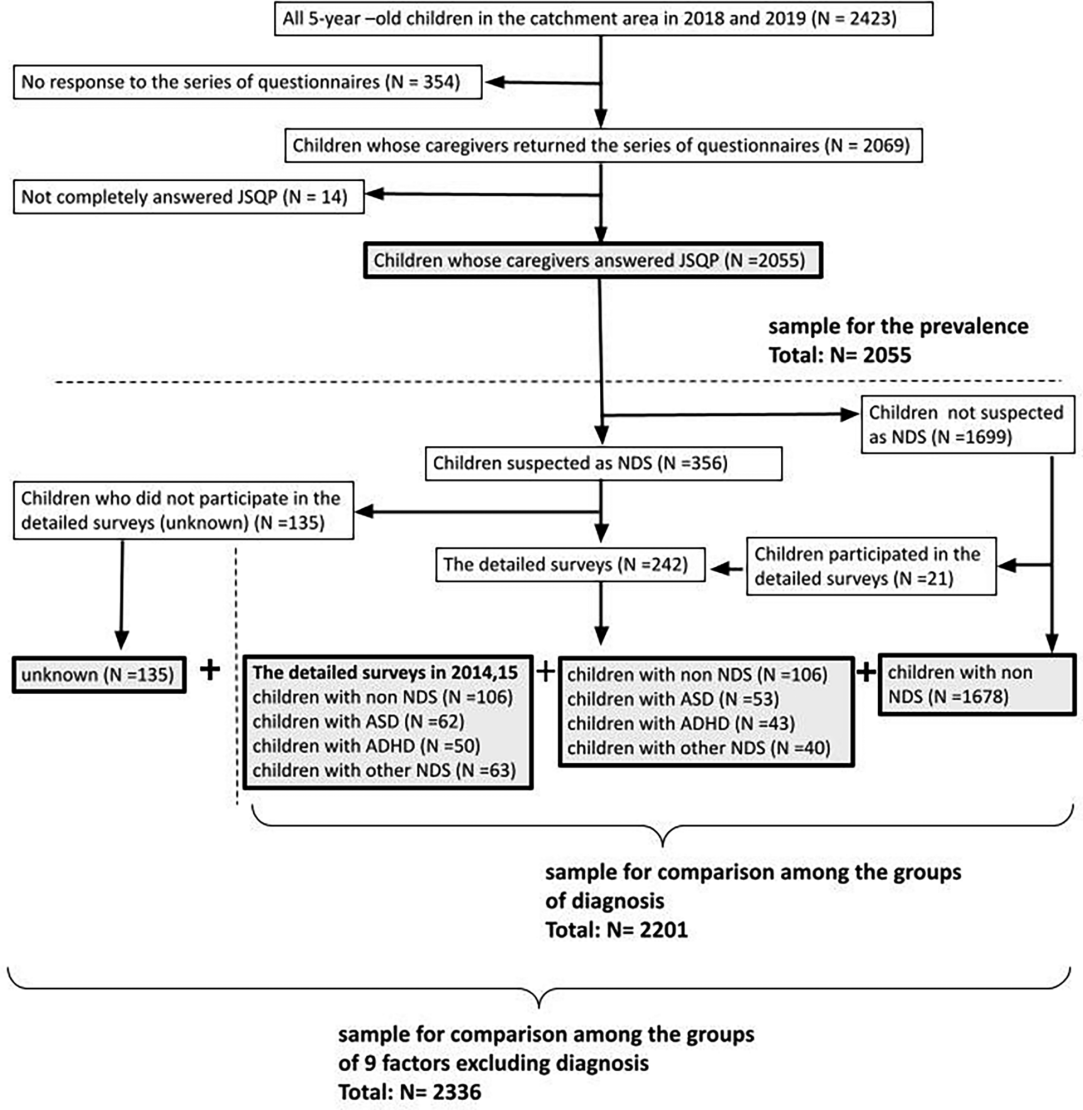
Sampling flow.

First, the data of 2,069 children was collected from the HFC study database. Their parents or caregivers responded to the first stage survey (response rate 85.4%), which targeted all 2,423 five-year-old children in the city in 2018 and 2019. Of these, excluding 14 children who did not respond to the sleep survey, 2,055 children (1,068 boys, 987 girls, response rate 84.8%) were analyzed for the prevalence of sleep problems (Refer to the area above the dotted line in Figure 1).

Next, among the 2,055 SC participants, 1,678 SC participants were defined as participants with non-NDS, excluding 21 children who participated in detailed examinations of the 1,699 children for whom NDS was not suspected in the primary survey. The diagnoses of 242 children who participated in detailed medical examinations were classified as ASD, ADHD without ASD (ADHD), and other neurodevelopmental disorders (other NDS). In addition, 135 children with suspected NDS but whose diagnosis was unknown because they did not participate in the detailed survey were excluded only in analyzes related to diagnosis. Details of the diagnostic groups of the 2,055 children are shown in Table 1.

**Table 1:** Diagnosis and details of participants in 2018 and 19/in 2014, 15, 18 and 19.

	Participants in 2018 and 19			Participants in 2014, 15, 18, and 19		
	Total	Male	Female	Total	Male	Female
	2,055	1,068	987	2,336	1,230	1,106
Participants diagnosed with non-NDS	1,784	914	870	1,890	963	927
Participants diagnosed with ASD	53	44	9	115	86	29
Participants diagnosed with ADHD exclude ASD	43	29	14	93	60	33
Participants diagnosed with other NDS	40	22	18	103	62	41
Unknown*	135	59	76	135	59	76
Month age	mean 61.45 (SD 1.734)	61.43 (1.700)	61.48 (1.770)	mean 61.47 (SD 1.723)	61.44 (1.705)	61.49 (1.744)

* Unknown: Children who did not participate in the detailed surveys.

Because the number of children diagnosed was small in the 2018 and 2019 data, to increase the sample size, 281 children were added who participated in detailed examinations in 2014 and 2015 and were diagnosed. They underwent the same detailed examinations in 2018 and 2019 and were diagnosed using the same procedure. With this addition, 2,201 participants (participants with non-NDS: 1,890, with ASD: 115, with ADHD: 93, with other NDS: 103 children) were used to analyze the correlation between sleep problems and NDS diagnosis. (Refer to the lower right area of the dotted line in Figure 1).

In addition, 135 (“unknown”) children were added who were excluded from the analysis of sleep problems and diagnoses. Then for 2,336 children the correlation between sleep problems and selected socio-demographic and lifestyle-related factors were analyzed (Refer to the bottom area of Figure 1).

### 2.4 Instruments and setting

#### 2.4.1 Japanese sleep questionnaire for preschoolers (JSQ-P)

JSQ-P is a questionnaire in which parents and caregivers answer questions about their children's sleep habits. It was developed by researchers in the Department of Pediatrics at Osaka University in 2010 as a tool to assess adequate sleep in Japanese children (24, 25).

The questionnaire consists of 39 sleep-related questions, answers scored on a 6-point scale, and some questions about sleep habits that are unrelated to scores [ex: average waking time and bedtime, daily TV/video viewing time (screen time), and the time from going to bed to falling asleep, etc.].

The 39 sleep-related questions are divided into 10 subcategories. The 10 subcategories include restless legs syndrome-sensory and -motor, obstructive sleep apnea, parasomnias, insomnia/circadian rhythm disorder, morning symptoms, daytime excessive sleepiness, daytime behaviors, sleep habits, and Insufficient sleep.

(Refer Supplementary Table B).

#### 2.4.2 Definition of sleep problems

The total score of the 39 questions on the JSQ-P was utilized to assess sleep problems. The cutoff value for sleep problems of JSQ-P total score is 86 points, which was created based on the diagnosis of sleep disorders in Japanese children as an outcome (24, 25). Thus, a score of 86 or higher is likely to indicate the presence of sleep problems. In our study, JSQ-P total score ≥86 was defined as having sleep problems.

#### 2.4.3 Contents of the 10 selected factors and each grouping

In order to examine various factors that may be correlated with sleep in 5-year-old children, 10 factors were selected from the HFC study database. First, these 10 factors were selected; “1 Diagnosis” of NDS based on the results of detailed examinations. Next, based on the epidemiological questions in the primary survey, we selected four socio-demographic factors: “2 Birth month”, “3 Childcare place during daytime”, “4 Annual family income”, and “5 Number of siblings”. Finally, based on the answers to the non-scored questions on the JSQ-P, we selected four factors to explain the child's lifestyle: “6 Bedtime”, “7 Waking time”, “8 Sleeping hours”, “9 Sleep onset delay” and “10 Screen time”. Sleeping hours were calculated from bedtime and waking time. Factors number 1,2,3,4,5, and 10 were selected because children with ASD, ADHD (14, 15), spring birth month (26), nursery school (27), low income (28–30), many siblings (31, 32), or long screen time (33) had poor sleep in previous studies. Furthermore, we added indicators of sleep habits such as bedtime, waking time, sleeping hours, and sleep onset delay.

In addition, each selected factor other than lifestyle was divided into groups according to diagnostic results, epidemiological survey options, and descriptive content, and lifestyle factors were divided into several groups based on time. Details are shown in Table 2.

**Table 2:** Selected factors, contents of questionnaires and groups.

Selected factors	Questionnaires	Groups
Diagnosis		Participants diagnosed with non-NDS, ASD (including other diagnosis), ADHD (exclude ASD), other NDS (DCD and ID exclude ASD or ADHD)
Birth month	Date of birth	4–6, 7–9, 10–12, 1–3 (month of birth)
Childcare places during daytime	Who are the main daytime caregivers of your child? 1. Nursery School 2. Kindergarten 3. Father 4. Mother 5. Grandfather 6. Grandmother	Nursery School, Kindergarten, Home
Annual family income	Which of the following applies to the total annual income of your child's home? 1. less than 2 million yen 2. 2–4 million yen 3. 4–7 million yen 4. 7–10 million yen 5. 10 or more million yen 6. unknown	Less than 2 (<2) million yen, 2–4 (2 ≤ <4) million yen, 4–7 (4 ≤ <7) million yen, 7–10 (7 ≤ <10) million yen, 10 or more (≥10) million yen (notation in Tables)
Number of siblings	How many siblings does your child have?	No siblings, one sibling, two or more siblings
Bedtime	AM/PM ():()	Earlier than 21:00 (<21:00), 21:00–21:59 (21:00 ≤ <22:00), 22:00—later (≥22:00) (notation in Tables)
Waking time	AM/PM ():()	earlier than 6:30 (<6:30), 6:30–6:59 (6:30 ≤ <7:00), 7:00–7:29 (7:00 ≤ <7:30), 7:30 or later (≥7:30) (notation in Tables)
Sleeping hours		shorter than 9 (<9) h, 9–9.5 (9 ≤ <9.5) h, 9.5–10 (9.5 ≤ <10) h, 10 (≥10) h or longer (notation in Tables)
Sleep onset delay	How long does it take for your child to fall asleep after getting into the bed? 1. shorter than 10 min 2. shorter than 20 min 3. shorter than 30 min 4. shorter than 1 h 5. 1 h or longer 6. unknown	shorter than 10 min (<10 min), shorter than 20 min (10 ≤ <20 min), shorter than 30 min (20 ≤ <30 min), shorter than 1 h (30 ≤ <1 h), 1 h or longer (≥1 h) (notation in Tables)
Screen time	How much time does your child spend watching TV or video? () h () min	shorter than 2 h (<2 h), 2–4 h (2 ≤ <4 h), 4 h or longer (≥4 h) (notation in Tables)

Since diagnoses and options of questionnaire were categories, groups were created based on them. Regarding bedtime, waking time, sleep onset delay, and screen time, because most of the answers were written in rough time formats, groups were created dividing by a certain time range.

For each selected factor, samples without responses to the questionnaire were excluded because they could not be assigned to a group.

### 2.5 Statistical analysis procedure

To determine the prevalence of sleep problems, the percentage of children with sleep problems out of 2,055 children in 2018 and 2019 was calculated.

In order to clarify if the selected factors associated with sleep problems, a chi-square test was conducted on the presence/absence of sleep problems and each selected factor group. In addition, to determine which groups had significantly more sleep problems, a residual analysis was performed between groups and compared using adjusted standardized residual coefficients. The significance level was *p* < 0.05 and significant adjusted standardized residual coefficient >1.96.

Furthermore, to examine the contribution of bedtime to sleeping hours and the contribution of screen time to bedtime, chi-square test was conducted between the two selected factors, and then conducted a residual test and compared them using adjusted standardized residual coefficients. The significance level was *p* < 0.05 and significant adjusted standardized residual coefficient >1.96.

SPSS ver. 26 was used as the calculation and analysis software.

### 2.6 Ethics statements

This study was approved by the Ethics Committee of Hirosaki University Graduate School of Health Science (approval number 2023-033). The studies were conducted in accordance with the local legislation and institutional requirements. Written informed consents for participation in this study were provided by the children's legal guardians.

## 3 Results

### 3.1 Overall

The prevalence of sleep problems of 5-year-old children was 18.0% (Table 3).

**Table 3:** The prevalence of sleep problems.

Sleep problems − JSQ-P total <86		Sleep problems + JSQ-P total ≥86		Total	
Number	%	Number	%	Number	%
1,686	82.0%	369	18.0%	2,055	100%

The percentages of children with sleep problems of these groups were significantly higher than the other groups in each selected factor; participants diagnosed with ASD and ADHD, annual family income less than two million yen (<2 million yen), no siblings, bedtime 22:00 or later (≥22:00), waking time 7:30 or later (≥7:30), less than 9 h (<9 h) of sleep per night and sleep onset delay over 30 min (30 ≤ <1 h and ≥1 h) (Table 4). Meanwhile, the percentages of children with sleep problems in these groups were significantly lower than the other groups in each selected factor; participants with non-NDS, annual income four to seven million yen (4 ≤ <7 million yen), two or more siblings, bedtime earlier than 22:00 (<21:00 and 21:00 ≤ <22:00), waking time earlier than 6:30 (<6:30), sleep onset delay shorter than 20 min (<10 min and 10 ≤ <20 min), and screen time shorter than 2 h (<2 h). There were no significant differences of the percentage of children with sleep problems between the groups in the factors of birth month and childcare places during daytime.

**Table 4:** Selected factors and their percentages for each group.

	Groups	Total	Sleep problems − JSQ-P total <86		Sleep problems + JSQ-P total ≥86			*p*
			Number	%	Number	%	Adjusted residual	
1. Diagnosis (*N* = 2,201)	Participants diagnosed with non-NDS	1,890	1,610	85.2%	280	**14.8%**	**−10.2**	**<0.001**
	Participants diagnosed with ASD	115	57	49.6%	58	**50.4%**	**9.2**	
	Participants diagnosed with ADHD (exclude ASD)	93	56	60.2%	37	**39.8%**	**5.5**	
	Participants diagnosed with other NDS	103	77	74.8%	26	25.2%	1.9	
2. Birth month (*N* = 2,298)	4–6	561	462	82.4%	99	17.6%	−1.2	0.401
	7–9	649	529	81.5%	120	18.5%	−0.7	
	10–12	565	447	79.1%	118	20.9%	1.0	
	1–3	523	414	79.2%	109	20.8%	0.9	
3. Childcare place during daytime (*N* = 2,277)	Nursery school	1,840	1,472	80.0%	368	20.0%	1.7	0.245
	Kindergarten	413	345	83.5%	68	16.5%	−1.6	
	Home	24	20	83.3%	4	16.7%	−0.3	
4. Annual family income (*N* = 2,130)	<2 million yen	174	121	69.5%	53	**30.5%**	**3.9**	**<0.001**
	2 ≤ <4 million yen	671	526	78.4%	145	21.6%	1.9	
	4 ≤ <7 million yen	843	708	84.0%	135	**16.0%**	**−3.0**	
	7 ≤ <10 million yen	299	251	83.9%	48	16.1%	−1.5	
	≥10 million yen	143	119	78.3%	24	21.7%	−0.8	
5. Number of siblings (*N* = 2,288)	No siblings	433	328	75.8%	105	**24.2%**	**3.0**	**<0.01**
	One sibling	1,194	961	80.5%	233	19.5%	0.5	
	Two or more siblings	661	562	85.0%	99	**15.0%**	**−3.2**	
6. Bedtime (*N*= 2,327)	<21:00	260	229	88.1%	31	**11.9%**	**−3.2**	**<0.001**
	21:00 ≤ <22:00	1,444	1,218	84.3%	226	**15.7%**	**−5.6**	
	≥22:00	623	432	69.3%	191	**30.7%**	**8.4**	
7 Waking time (*N* = 2,332)	<6:30	586	516	88.1%	70	**11.9%**	**−5.2**	**<0.001**
	6:30 ≤ <7:00	752	608	80.9%	144	19.1%	−0.1	
	7:00 ≤ <7:30	691	549	79.5%	142	20.5%	1.0	
	≥7:30	303	210	69.3%	93	**30.7%**	**5.4**	
8. Sleeping hours (*N* = 2,291)	<9 h	462	345	74.7%	117	**25.3%**	**3.6**	**<0.01**
	9 ≤ <9.5 h	709	586	82.7%	123	17.3%	−1.6	
	9.5 ≤ <10 h	584	484	82.9%	100	17.1%	−1.6	
	≥10 h	536	433	80.8%	103	19.2%	−0.1	
9. Sleep onset delay (*N* = 2,304)	<10 min	629	538	85.5%	91	**14.5%**	**−3.5**	**<0.001**
	10 ≤ < 20 min	706	591	83.7%	115	**16.3%**	**−2.3**	
	20 ≤ < 30 min	640	520	81.3%	120	18.8%	−0.3	
	30 ≤ < 1 h	261	178	68.2%	83	**31.8%**	**5.5**	
	≥1 h	68	35	51.5%	33	**48.5%**	**6.2**	
10. Screen time^a^ (*N* = 2,291)	<2 h	822	687	83.6%	135	**16.4%**	**−2.7**	**<0.05**
	2 ≤ < 4 h	1,183	938	79.3%	245	20.7%	1.6	
	≥4 h	286	221	77.3%	65	22.7%	1.5	

^a^ Screen time: TV/video viewing hours.

The bold values mean significant value.

### 3.2 Diagnosis

In terms of the factor of diagnosis, the group of participants diagnosed with ASD and the group of participants diagnosed with ADHD had significantly higher percentage of sleep problems than the other groups, and the group of participants diagnosed with non-NDS had significantly lower than the other groups. The percentage of sleep problems was 50.4% in participants diagnosed with ASD, 39.8% in participants diagnosed with ADHD, 25.2% in participants diagnosed with other NDS (DCD and ID), and 14.8% in participants diagnosed with non-NDS. In other words, the prevalence of sleep problems in participants diagnosed with ASD was 3.4 times higher than the prevalence in participants with non-NDS, and the prevalence of sleep problems in participants diagnosed with ADHD was 2.7 times higher than the prevalence in participants with non-NDS.

### 3.3 Annual family income

In terms of the factor of annual family income, the group of less than two million yen (30.5%) had significantly higher percentage of children with sleep problems than the other groups and the group of four to seven million yen (16.0%) had significantly lower percentage than the other groups.

### 3.4 Number of siblings

In terms of the factor of number of siblings, the group without siblings (24.2%) had significantly higher percentage of children with sleep problems than the other groups and the group of two or more siblings (15.0%) had significantly lower percentage than the other groups.

### 3.5 Bedtime and waking time

In terms of the factor of bedtime, the group of 22:00 or later (30.7%) had significantly higher percentage of children with sleep problems than the other groups and two groups of earlier than 21:00 (11.9%) and 21:00–22:00 (15.7%) had significantly lower than the other groups.

In terms of the factor of waking time, the group of 7:30 or later (30.7%) had significantly higher percentage of sleep problems than the other groups and the group of earlier than 6:30 (11.9%) had significantly lower than the other groups.

### 3.6 Sleeping hours

In terms of the factor of sleeping hours, the group of shorter than 9 h (25.3%) had significantly higher percentage of children with sleep problems than the other groups. However, the group of longer sleeping hours did not result in significantly fewer sleep problems. In terms of the contribution of bedtime to sleep hours, children in group of bedtime earlier than 21:00 had significantly higher percentage of children in the group of 10 sleeping hours (67.1%), in group of bedtime 21:00–22:00 had significantly higher percentage of children in the groups of 9.0–9.5 sleeping hours (34.6%) and 9.5–10.0 sleeping hours (30.4%) respectively, and in group of bedtime over 22:00 had significantly higher percentage of children in the groups of less than 9.0 sleeping hours (48.1%) (Table 5).

**Table 5:** Bedtime and sleeping hours.

		Sleeping hours												Total	
		<9 h			9 h ≤ <9.5 h			9.5 h≤ <10h			10 h≤				
		*n*	%	Adjusted residual	*n*	%	Adjusted residual	*n*	%	Adjusted residual	*n*	%	Adjusted residual	*n*	*p*
Bedtime	<21:00	4	1.6%	**−7.9**	13	5.1%	**−9.5**	67	26.3%	0.3	171	**67.1%**	**17.5**	255	<0.001
	21:00≤<22:00	160	11.3%	**−13.5**	490	**34.6%**	**4.8**	431	**30.4%**	**6.9**	336	23.7%	0.5	1,417	
	22:00≤	298	**48.1%**	**20.3**	206	33.3%	1.5	86	13.9%	**−7.8**	29	4.7%	**−12.9**	619	
Total		462	20.2%		709	30.9%		584	25.5%		536	23.4%		2,291	

The bold values mean significantly higher percentage, significant adjusted residual.

### 3.7 Sleep onset delay

In terms of the factor of sleep onset delay, the group of more than 30 min and shorter than 1 h (31.8%) and one hour or longer (48.5%) had significantly higher percentage of children with sleep problems than the other groups, and the group of shorter than 10 min (14.5%) and more than 10 min and less than 20 min (16.3%) had significantly lower percentage of children with sleep problems than the other groups respectively.

### 3.8 Screen time

In terms of the factor of screen time, the group of shorter than 2 h (16.4%) had significantly lower percentage of children with sleep problems than the other group. In terms of the contribution of screen time to bedtime, children in the group of shorter than 2 h screen time had significantly higher percentage of children in the group of bedtime earlier than 21:00 (14.2%) or 21:00–22:00 (66.1%) respectively. The group of longer than 4 h screen time had a significantly higher percentage of children in the groups of 22:00 or later (41.9%) (Table 6).

**Table 6:** Screen time and bedtime.

	** **	Bedtime									Total	
		<21:00			21:00 ≤ < 22:00			22:00≤				
		*n*	%	Adjusted residual	*n*	%	Adjusted residual	*n*	%	Adjusted residual	number	*p*
Screen time	<2 h	116	**14.2%**	**3.6**	541	**66.1%**	**2.8**	162	19.8%	**−5.6**	819	<0.001
	2h ≤ < 4 h	116	9.8%	−1.9	736	62.3%	0	330	27.9%	1.3	1,182	
	4 h≤	19	6.7%	**−2.5**	146	51.4%	**−4.0**	119	**41.9%**	**6.2**	284	
Total		251	11.0%		1,423	62.3%		611	26.7%		2,285	

The bold values mean significantly higher percentage, significant adjusted residual.

## 4 Discussion

### 4.1 Population-based prevalence of sleep problems in 5-year-olds

In this study, the recent population-based prevalence of sleep disorders among 5-year-old children in Japan was 18.0%. This study is the first report in Japan to calculate prevalence on a population basis, using a sleep scale that is appropriate for Japanese culture and age, and limited to 5-year-old preschoolers. There were two previous reports with similar results to this result. Mindell et al. (8) reported that 20%–25% of school-aged and adolescent children had sleep problems, and Takeshima et al. reported that the prevalence of sleep disorders was 18.3%.

On the other hand, some previous studies reported higher prevalence rates than our results. Takahashi et al. reported that approximately 80% of 5-year-old children had sleep problems. His study used the same database as ours and surveyed a different group of children than our study population, using the Children's Sleep Habits Questionnaire (CSHQ) as a sleep measure. However, when using the CSHQ, the prevalence tends to be higher in countries with a culture of co-sleeping than in Europe and the United States (11). Furthermore, CSHQ does not set a cutoff value for sleep problems in Japanese children and uses Western standards. To calculate accurate prevalence rates, it is desirable to use at least a standardized scale in Japan. Although the JSQ-P was created based on the CSHQ, its reliability and validity have been confirmed because it was created using the diagnosis of sleep disorders in Japanese children as an outcome (24, 25).

Several meta-analyses have also reported higher prevalence rates of sleep problems in populations including preschoolers, such as 54% (12) and 38.9% (13). These results show that the prevalence varies depending on the sleep measures used and the definition of sleep problems.

Therefore, although a prevalence of 18% was obtained in this study, the possibility of a higher prevalence should be considered if the scale or the definition of sleep problems is changed.

### 4.2 Correlation between sleep problems and NDS diagnosis

Similar to previous studies, our results showed that participants diagnosed with ASD and participants diagnosed with ADHD had a higher prevalence of sleep problems (16). In Singh et al.'s systematic review, compared to the prevalence of sleep problems in children with non-NDS, the prevalence in children with ASD was 2–3 times higher (16). In our study, compared to the prevalence of sleep problems in participants with non-NDS, the prevalence in participants with ASD was 3.4 times higher, which was slightly higher than previous reports. Due to the small number of children with NDS when investigating the association between NDS and sleep problems, we added the number of 2-year NDS groups to increase the power of the sample. The prevalence of sleep problems in children with ASD may be higher than expected, although this study cannot be compared with other studies as there are no studies limited to 5-year-old preschool children. Additionally, compared to the prevalence of sleep problems in children with non-NDS, the prevalence in participants with ADHD was 2.7 times higher. These results are the ratio of the prevalence of sleep problems in children with ASD and children with ADHD to that in children with non-NDS, examined within a single population. This is the first report in Japan to clarify the relative risks of sleep problems in children with ASD and children with ADHD.

### 4.3 Correlation between sleep problems and socio-demographic and lifestyle related factors

Similar to our results, in terms of annual family income, previous studies have found that low-income groups have more sleep problems (28–30). On the other hand, in the Grandner et al. (34) study, people in low-income groups reported fewer sleep problems after controlling for a number of covariates. They investigated the correlation between adults' own annual income and sleep but noted that there may be no correlation between low income and sleep problems. It is possible that our study would not yield similar results if we similarly adjusted for covariates. Additionally, the prevalence of sleep problems is not significantly lower among children in the highest income groups, so other factors should be considered in the correlation between annual family income and children's sleep problems. Differences in percentages between income groups in this study may be due to more direct factors, such as the work styles and lifestyles of parents and caregivers, rather than just annual family income. However, these direct factors were not investigated in this study.

The higher prevalence of sleep disorders in children without siblings may be due to the sleeping habits of the person who sleeps with the child. Previous studies have reported that children who typically sleep with siblings are more likely to have sleep problems (31, 32). These studies, conducted in a different culture than Japan, showed that children who shared a bed with family members were more likely to have sleep problems than children who slept alone. Traditionally in Japan, preschoolers share a bedroom with their parents and siblings. Children with siblings sleep with their siblings, and children without siblings sleep with their parents. Because Japanese adults tend to sleep less, it is thought that children are likely to be influenced by the lifestyle of adults who co-sleep. This means that children who sleep with their parents are more likely to have sleep problems than children who sleep with their siblings. In Japanese culture, it was suggested that sleeping with siblings may have a more positive impact on preschoolers' sleep habits.

In terms of children's sleeping hours, the American Academy of Sleep Medicine recommends 10–13 h of sleep for 3–5-year-olds (35). A meta-analytic study reported that the average sleeping hours for 4- to 5-year-olds was 11.5 h (9.1–13.9 h) (36). In our study, only 23.4% of 5-year-olds slept longer than 10 h (Table 5), and many of the participants fell below the recommended and average sleeping hours. Sleeping hours, which was one of the selected factors, was calculated from the bedtime and waking time answered by parents, so it represents sleeping hours only at night. 95% of the participants were enrolled in nursery schools or daycare centers, where children took a 1–2 h nap each day. Therefore, if sleeping hours are taken into account, including nap time, the daily sleeping hours would be 1–2 h longer than this result. It was also found that children who went to bed earlier and woke up earlier had fewer sleep problems, and it was observed that the later the bedtime group, the greater the percentage of children who slept less. The results may support common sense recommendations to go to bed early and wake up early.

Sleep onset delay may be due to a circadian rhythm disorder, and behavioral therapy such as sleep preparation and oral melatonin intake may be effective. A previous study reported that the average sleep latency for 3- to 5-year-olds was 17.4 min, and the average sleep latency for 5- to 6-year-olds was 16 min (36). Considering this case as well, it seems appropriate for preschool children to fall asleep within 30 min for a good night's sleep.

The association of sleep problems and screen time/presence of bluelight has been reported in previous studies. For example, blue light suppresses melatonin secretion (37, 38). Additionally, excessive screen time in children is associated with teeth grinding during sleep (39). A recent meta-analysis also showed that frequent use of social media is a risk factor for sleep deprivation in young people (40). Additionally, our results showed that children who spend more screen time tend to go to bed later. A meta-analysis showed an association between excessive screen time and delayed bedtime in children (33). When children get home late from nursery school and spend more time on screen, their bedtimes are later. In this study, children with less than 2 h of screen time had a lower prevalence of sleep problems, so we recommend limiting screen time to a maximum of 2 h.

Sleep is particularly important for children who are still developing, as it is related to cognitive function, physical and mental health, and daytime behavior (5, 7, 40, 41). It may be that children's daytime troubles, anxiety, depression, or obesity are related to sleep. However, sleep problems are complex, and it is unclear whether they are directly related to daytime behavior or health problems.

It is important to investigate children's sleep problems in detail and fundamentally solve each problem. However, we should first work on improving our children's sleep by adjusting their environment and lifestyle. Previous studies have shown that children's sleep can be improved through simple environmental and lifestyle approaches. Yoshizaki et al. reported that an interactive smartphone app that provides suggestions tailored to culture and family life was effective in improving sleep habits in Japanese children (42). Additionally, Halal et al. have found that behavioral approaches, including providing written information, were effective in educating children about sleep, and that interventions in the child's environment were also effective in maturing the sleep process (43). To solve children's sleep problems, it would be better if their families knew about lifestyle habits that are good for children's sleep from an early stage of development.

### 4.4 Limitations

This study has some limitations. First, in this study, because this study was a cross-sectional survey, it is not possible to state causal relationships. The prevalence of sleep problems within each selected factor was analyzed using chi-square analysis, and only univariate analysis were performed, so confounders and other explanatory variables were not considered. Therefore, there might be significant differences in factors that were not actually related to sleep problems.

Second, one of the JSQ-P question items is “Sleep later than 10 p.m.”, and the score of this item is included in the JSQ-P total score. Therefore, the bedtime group and the JSQ-P total score are not strictly independent. However, since it was only one item out of 39, the effect was considered to be small, and the correlation between the Bedtime group and the JSQ-P total score were analyzed.

Third, the sleep scale used in this study was created to suit the Japanese population, and other sleep scales have different definitions of sleep problems, so it is not possible to strictly compare past studies with this study. Because sleep measures vary across studies, a systematic review would be more meaningful if standards were standardized.

Finally, the findings of this research suggest several recommendations: (1) 1 in 5–6 children aged 5 years have sleep problems. Therefore, we should always expect a certain number of children to have sleep problems during health checkups for preschoolers. (2) In cultures that traditionally sleep with family members, sleeping with siblings doesn't necessarily have a negative effect on sleep, but can actually have a positive effect. (3) To reduce sleep problems in children, we recommend the following four lifestyle habits: going to bed earlier than 22:00, waking up at 6:30 (or before 7:30 at the latest), falling asleep for less than 20 min (at least 30 min for her), and screen time less than 2 h. (4) In future studies, when analyzing the association between NDS such as ASD and ADHD and sleep problems, confounding factors such as socio-demographic or lifestyle-related factors should be appropriately considered.

## 5 Conclusion

This study showed the population-based prevalence of sleep problems among 5-year-old children in Japan. Using the JSQ-P, a sleep scale appropriate for Japanese children, the findings report 18% prevalence rate of sleep problems in 5-year-old children. Furthermore, the findings revealed sociodemographic and lifestyle-related factors besides ASD and ADHD that may be associated with sleep problems. These results recommend that children should go to bed early and wake up early and have less than 2 h of screen time. This study suggested several factors and specific recommendations for intervening in sleep problems. We hope that these results will be considered as one of the basis for sleep intervention.

## Data Availability

The raw data supporting the conclusions of this article will be made available by the authors, without undue reservation.
