# Supplementary material for: Prevalence and factors of sleep problems among Japanese children: a population-based study
Source: Front Pediatr. 2024 Apr 4;12:1332723. doi: 10.3389/fped.2024.1332723 (PMC11024267; doi:10.3389/fped.2024.1332723)
Supplement: Supplementary file 1 [file Table1.docx]

Supplementary Material

# Supplementary Figures and Tables

Table A: Questionnaires and Examinations used in the HFC study

Table. B: JSQ-P subscales and JSQ-P items

## Supplementary Tables

**Supplementary Table A:** Questionnaires and Examinations used in the HFC study

| **Questionnaires at the primary survey** | |
| --- | --- |
| Questionnaire of demographic information | The demographic information includes birth month, childcare place during daytime, annual income and siblings. |
| Autism Spectrum Screening Questionnaire (ASSQ) | It consists of 27 items that ask whether the child is applied to typical characteristics of autism in term of social, language, behavioral, and interest. (Adachi M, et al. 2018) |
| Strengths and Difficulties Questionnaire (SDQ) | It consists of 25 items on five subscales: behavior, hyperactivity, emotionality, peer relationships, and prosociality. The total score reveals the necessity of support. (Matsuishi, et al. 2008) |
| ADHD-Rating Scale-IV (ADHD-RS-IV) | It consists of 18 items that evaluate attention, hyperactivity and impulsivity. ( Takayanagi, et al.2016) |
| Developmental Coordination Disorder Questionnaire (DCDQ) | It consists of 15 items that evaluate control during movement, fine motor and general coordination. (Nakai, et al. 2011) |
| Japanese version of the Parenting Stress Index (PSI) | It consists of 78 questions to evaluate parenting stress over two domains: the child domain (38 questions) and the parent domain (40questions). In this study, only child domain was conducted. (Narama, et al. 1999) |
| Children were considered “screen positive” if one of these criteria (a)–(d) was met:  a) Parent-rated ASSQ scores ≧ 19  *Sensitivity: 0.552, specificity: 0.994 for diagnose of ASD  b) PSI scores > 75% percentile of PSI score distributions  *Accuracy of ASD: .92, ADHD: .90, DCD: .82  (These accuracies were evaluated by AUC of ROC.)  c) (ASSQ≧9) and [two or more items from (ADHD-RS-total≧M19/F14, ADHD-RS-inattention≧M12/F9, ADHD-RS-hyperactivity/impulsively≧M8/F5)]  d) (SDQ≧M18/F13) and [one item from (ASSQ≧9, ADHD-RS-total≧M19/F14, ADHD-RS-inattention≧M12/F9, ADHD-RS-hyperactivity/impulsively≧M8/F5, DCDQ-total≦M36/F40, DCDQ-control during movement≦M14/F14, DCDQ-fine motor and handwritings≦M8/F10 or DCDQ-general coordination≦M11/F13)]  * Accuracy of ASD: M97.6/F98.2%, ADHD: M94.8/F95.2%, DCD: M96.6/F100%  (These accuracy rates were evaluated by logistic regression analysis) | |
| **Examinations for comprehensive assessment at the detailed survey** | |
| Diagnostic Interview for Social and Communication Disorders (DISCO) | A semi-structured interview schedule designed to collect information on development and behavior (Wing, et al. 2002 ) |
| Japanese version of the Wechsler Intelligence Scale for Children, 4th edition (WISC-IV) | A examination that evaluate intelligence of children between the ages of 6 and 16 (Wechsler, 2010) |
| Movement Assessment Battery for Children, 2nd edition (MABC-2) | Assessment for motor impairments of children in children aged 3–16 years (Henderson, et al. 2007) |
| Autism Diagnostic Observation Schedule 2nd edition (ADOS-2) | An examination for definitive diagnosis of ASD (Lord, et al. 2012) |

[Reference]

Adachi M, Takahashi M, Takayanagi N, Yoshida S, Yasuda S, Tanaka M et al. Adaptation of the Autism Spectrum Screening Questionnaire (ASSQ) to preschool children. Hadjikhani N, editor. PLOS ONE. 2018 Jul 10;13(7): e0199590. DOI: 10.1371/journal.pone.0199590.

Henderson SE, Sugden DA, Barnett A. The movement assessment battery for children. 2nd ed. London: The Psychological Corporation; 2007.

Lord C, Rutter M, Dilavore PC, Risi S, Gotham K, Bishop LS. The Autism Diagnostic Observation Schedule 2nd Edition (ADOS-2). Western Psychological Services: Los Angeles, CA; 2012.

Matsuishi T, Nagano M, Araki Y, Tanaka Y, Iwasaki M, Yamashita Y et al. Scale properties of the Japanese version of the Strengths and Difficulties Questionnaire (SDQ): a study of infant and school children in community samples. Brain and Development. 2008;30(6):410–5. DOI: 10.1016/j.braindev.2007.12.003.

Nakai A, Miyachi T, Okada R, Tani I, Nakajima S, Onishi M et al. Evaluation of the Japanese version of the Developmental Coordination Disorder Questionnaire as a screening tool for clumsiness of Japanese children. Res Dev Disabil. 2011;32(5):1615–22.

Narama M, Kanemasu Y, Araki A, Maru M, Nakamura N, Takeda J et al. Validity and reliability of Japanse version of the Parenting Stress Index [in Japanese]. J Child Health. 1999;58(5):610–6.

Takayanagi N, Yoshida S, Yasuda S, Adachi M, Kaneda-Osato A, Tanaka M et al. Psychometric properties of the Japanese ADHD-RS in preschool children. Res Dev Disabil. 2016;55:268–78. DOI: 10.1016/j.ridd.2016.05.002.

Wing L, Leekam SR, Libby SJ, Gould J, Larcombe M. The diagnostic interview for social and communication disorders: background, inter-rater reliability and clinical use. J Child Psychol Psychiatry. 2002;43(3):307–25.

Wechsler D. Japanese WISC-IV Publication Committee. Japanese version of the Wechsler intelligence scale for children-fourth edition. Nihon Bunka Kagakusha: Tokyo, Japan; 2010.

**Supplementary Table. B:** JSQ-P subscales and JSQ-P items

| **JSQ-P　subscales** | **Questions** |
| --- | --- |
| RLS^*^-sensory | Q14 Says legs hurt at night |
|  | Q15 Says legs feel hot at night |
|  | Q16 Says legs feel strange at night |
| RLS^*^-motor | Q17 Rubs feet at night |
|  | Q18 Touches feet at night |
| OSA^**^ | Q11 Moves a lot during the night |
|  | Q29 Clenches teeth |
|  | Q30 Sleeps with mouth open |
|  | Q31 Sleeps with head arched back |
|  | Q32 Snores loudly |
|  | Q33 Stops breathing |
|  | Q34 Snorts and gasps |
| Morning symptoms | Q1 Grumpy in the morning |
|  | Q2 Needs a long time to wake up |
|  | Q3 Takes a long time to get out of bed |
| Parasomnias | Q25 Cries at night |
|  | Q26 Wakes screaming and cannot be calmed down |
|  | Q27 Woken by scary dreams |
|  | Q28 Wakes up at any little sound |
|  | Q37 Wakes more than once during the night |
| Insomnia/Circadian rhythm disorder | Q4 Late for nursery school or kindergarten due to waking up late |
|  | Q5 Gets more than 2 naps |
|  | Q12 Snoozes at nursery school or kindergarten |
|  | Q13 Goes to bed after 10 pm |
|  | Q19 Gets excited at night |
|  | Q20 Gets grumpy at night |
|  | Q22 Taken for a car ride due to sleeping difficulty |
|  | Q23 Has trouble going to sleep |
|  | Q38 Has no pattern to sleep and wake-up times |
|  | Q39 Day–night reversal |
| Daytime excessive sleepiness | Q6 Falls asleep during the daytime |
|  | Q9 Seems sleepy in the daytime |
|  | Q10 Looks run-down in the daytime |
| Daytime behaviors | Q7 Restless in the daytime |
|  | Q8 Has poor concentration in the daytime |
| Sleep habit | Q21 Sleeps without being tucked in |
|  | Q24 Goes to bed by him/herself |
| Insufficient sleep | Q35 Stays up later than usual by an hour or more the day before a holiday |
|  | Q36 Wakes up more than an hour later on a holiday |

*RLS; restless legs syndrome

**OSA; obstructive sleep apnea

[Reference]

Hirata I, Mohri I, Kato-Nishimura K, Tachibana M, Kuwada A, Kagitani-Shimono K et al. Sleep problems are more frequent and associated with problematic behaviors in preschoolers with autism spectrum disorder. Res Dev Disabil. 2016 Feb-Mar:49-50:86-99. DOI: 10.1016/j.ridd.2015.11.002.
